# Clinic-based characteristics of animal-related injury presentations in Hangzhou, China: a 10-year retrospective analysis

**DOI:** 10.3389/fpubh.2026.1825693

**Published:** 2026-05-26

**Authors:** Jing Chen, Yanna Le, Hua Qin, Wanli Yang, Xue Hu, Weiwei Cai, Jing Wang

**Affiliations:** 1 Department of General Medicine, Hangzhou Occupational Disease Prevention and Control Hospital, Hangzhou, China; 2 Department of Infectious Disease Control and Prevention, Hangzhou Center for Disease Control and Prevention (Hangzhou Health Supervision Institution), Hangzhou, China

**Keywords:** animal-related injuries, epidemiology, injury prevention, public health, wound management

## Abstract

**Background:**

Animal-related injuries are a significant public health concern, particularly in urban settings with high population density and widespread pet ownership.

**Methods:**

This retrospective descriptive study was based on a clinic-based case series of animal-related injury presentations at a high-volume rabies vaccination clinic in Hangzhou, China, from 2015 to 2024. Demographic characteristics, injury site, exposure category, animal species, and time to presentation were extracted from outpatient records. Delayed presentation was defined as seeking care more than 3 days after injury. Descriptive analyses were performed to summarize clinic-based patterns. Multivariable logistic regression was used to identify factors associated with delayed presentation and to examine differences between dog- and cat-related injuries.

**Results:**

A total of 193,849 cases were included. Injuries exhibited significant seasonality, with the highest incidence in summer (30.16%) and the lowest in winter (20.22%). Females accounted for 55.9% of cases, and the 16–30 age group was the most affected (36.2%). Upper extremities were the predominant injury site (61.1%), and most cases were WHO Category II exposures (83.8%). Domestic animals caused 82.5% of injuries, with dogs (65.85%) and cats (27.64%) being the main offending animals. The rate of delayed presentation (>3 days) increased from 10.48% in 2015 to a peak of 20.67% in 2019 and then stabilized at approximately 16%. Multivariable logistic regression analysis showed that both Category I and Category III exposures (compared with Category II), injuries caused by non-dog species, and injuries caused by non-domestic animals were associated with higher odds of delayed presentation, while younger individuals and those with head and face injuries were less likely to delay care-seeking. For cat-related injuries (vs. dog-related injuries), independent predictors included increasing age, female sex, head and face injuries, and Category III exposure.

**Conclusion:**

This study provides a comprehensive description of animal-related injury presentations at a major urban rabies vaccination clinic. The findings highlight variation in healthcare-seeking behavior and injury patterns across demographic and exposure characteristics. These results may inform future hypothesis-driven research and support the development of targeted public health education strategies.

## 1 Introduction

Animal-related injuries represent a significant global public health concern, causing not only direct physical trauma but also serving as the primary transmission route for rabies and other fatal zoonotic diseases. According to statistics from the World Health Organization (WHO), approximately 59,000 people die from rabies annually worldwide, with 99% of human cases transmitted by dogs (1). Rabies is virtually always fatal once clinical manifestations develop, ranking among the most lethal infectious diseases worldwide (1, 2). The disease burden is predominantly concentrated in developing countries in Africa and Asia, placing substantial strain on public health systems (2, 3). Post-exposure prophylaxis (PEP) constitutes the cornerstone of rabies prevention; prompt and appropriate wound care, together with timely immunization, significantly reduces the risk of fatal outcomes (1, 4).

Beyond the risk of rabies transmission, animal-related injuries can result in various bacterial infections, tetanus, and other complications, generating significant morbidity and economic losses (5, 6). Drawing on data from the Global Burden of Disease Study, Zuo et al. (7) revealed pronounced global disparities in rabies incidence and mortality between 1990 and 2021—particularly highlighting the disproportionate impact on low-income nations. Chen et al. (8) further revealed trends in global and Chinese rabies burden and provided forecasts to 2040. Consequently, gaining a thorough understanding of the epidemiological profile of animal-related injuries is vital to inform evidence-based prevention and intervention policies.

Worldwide, the incidence and nature of animal-related injuries differ markedly by region—shaped by a range of contextual determinants including livestock management systems, climate, socioeconomic status, and the scope and quality of public health initiatives. Multiple studies have demonstrated that environmental factors, including climate and temperature, are closely associated with the incidence of animal-related injuries. Gautam et al. (9), using artificial intelligence-based analysis, showed that climate and air pollution are significant determinants of dog-related injury incidence. Research conducted by Yılmaz et al. (10) and Zeng et al. (11) consistently revealed marked variability in how temperature influences animal-related injury incidence—across time, geography, and demographic subgroups.

China was previously among the countries with the highest rabies burden. Through large-scale dog vaccination and PEP promotion, China has achieved substantial progress (12). Research by Wang et al. (13) on advances in adjuvanted rabies vaccines indicates that novel vaccines provide new options for rabies prevention. However, China continues to face considerable challenges in controlling animal rabies, including insufficient dog vaccination coverage, difficulties in managing stray animals, and inadequate public awareness regarding PEP (14). Therefore, continuous epidemiological surveillance and research remain crucial.

In recent years, multiple epidemiological studies on animal-related injuries have been conducted across China. Wang et al. (15) reported an incidence rate of 752.1 per 100,000 for animal-related injuries in Sandun Town, Hangzhou, significantly higher than the national average. A study by Shi et al. (16) in Beijing’s Haidian District (2011–2020) demonstrated a distinct summer peak in animal-related injuries. Research from Beijing’s Fangshan District (2020–2023) similarly found a higher proportion of female patients, contrasting with the male predominance reported in most international studies (17). The study by Ren et al. (18) on human rabies in Zhejiang Province provided important insights into the disease burden in this region.

These studies have revealed common characteristics of animal-related injuries in China: dogs are the primary injuring animals and the main source of rabies transmission; the upper extremities are the most frequently injured sites (19); injury incidence demonstrates distinct seasonal variations, peaking in summer (10, 11, 20); and significant regional variations exist in epidemiological patterns, reflecting the influence of geographic, climatic, and animal husbandry factors.

In addition to epidemiological characteristics, post-injury healthcare-seeking behaviors represent an important aspect of research. Liu et al. (21) found that animal bite victims in China often exhibit improper wound management and delays in PEP initiation, with educational level and socioeconomic factors significantly influencing proper wound management and timely medical presentation. El Bazi et al. (22) further identified patient age, residential setting (urban versus rural), bite severity and wound characteristics, and dog-related exposure as independent correlates of delayed PEP initiation. These findings underscore the necessity of strengthening community-level health literacy regarding rabies risk and evidence-based PEP management protocols.

Regarding clinical management, the trauma burden of animal bites should not be overlooked. Ali et al. (23) described the epidemiological characteristics of animal bites presenting to a pediatric emergency department. Ng et al. (24) assessed the surgical burden of animal bites referred to orthopedics. Price et al. (25) conducted a retrospective study of social and environmental predictors for dog-related injuries in Wales. These studies provide important perspectives for understanding the clinical impact of animal-related injuries.

Despite the substantial body of existing research, several gaps and limitations persist. First, long-term, large-scale studies are relatively scarce, with most research limited by small sample sizes or short study durations. Second, there is a lack of detailed comparative analyses of injury characteristics by animal species, particularly differences between cats and dogs, the two most common sources of injury. Third, specialized analyses focusing on injuries caused by rabid or suspected rabid animals are limited. Additionally, with China’s ongoing urbanization and rising pet ownership rates, animal-related injury patterns in urban areas may be evolving, necessitating more comprehensive data from urban populations to update our understanding of this issue.

Hangzhou is an economically developed city on China’s eastern coast, where pet ownership rates have continued to rise in recent years. Hangzhou Occupational Disease Prevention and Control Hospital used to operate the largest rabies vaccination clinic in the city and has maintained comprehensive records over a 10-year period (2015–2024). This study encompasses 193,849 animal-related injury cases, making it one of the largest single-center epidemiological studies of animal-related injuries in urban China to date.

This study aims to describe the characteristics of animal-related injury presentations at a major rabies vaccination clinic in Hangzhou from 2015 to 2024, with a focus on demographic patterns, injury characteristics, and healthcare-seeking behavior. Additionally, we explore factors associated with delayed presentation and differences between dog- and cat-related injuries using multivariable analysis.

## 2 Materials and methods

### 2.1 Research design and data sources

This was a retrospective descriptive study based on a clinic-based case series, collecting data on animal-related injuries recorded in the outpatient registration system of the Hangzhou Occupational Disease Prevention and Control Hospital from January 2015 to December 2024. Hangzhou Occupational Disease Prevention and Control Hospital previously operated one of the largest rabies vaccination clinics in Hangzhou.

The study population comprised all individuals who presented to the Hangzhou Occupational Disease Prevention and Control Hospital for clinical evaluation or management of animal bite injuries between January 1, 2015 and December 31, 2024. Inclusion criteria were: (1) a documented history of exposure to an animal with potential to cause injury; (2) with available outpatient records including key variables such as exposure classification, injury site, and time to presentation; and (3) receipt of on-site medical evaluation, treatment, or post-exposure prophylaxis (PEP) counseling at the hospital.

### 2.2 Data extraction

All data from cases presenting to the rabies vaccination clinic were extracted based on visit date. The extracted variables included WHO exposure category, demographic characteristics (gender, age), injury site, wound management, time from injury to clinic presentation (days), and animal category analysis (animal ownership status, injurious animal species). The data represent patients presenting to a single high-volume rabies vaccination clinic and do not capture all animal-related injuries in the general population. Detailed data on post-exposure prophylaxis (PEP) were not available; therefore, time to presentation was used as a proxy indicator of healthcare-seeking behavior.

### 2.3 Measurement variables

Age was categorized into five groups: ≤15 years, 16–30 years, 31–45 years, 46–60 years, and >60 years. Anatomic injury sites were classified as head/face/neck, upper extremities, trunk, lower extremities, and other (e.g., genitalia or multiple sites). Wound management categories were defined based on patient-reported actions prior to and/or at the clinic visit, including self-treatment, clinic treatment, both, or no treatment. These categories reflect care-seeking behavior rather than the quality or adequacy of wound management, as detailed information on the specific procedures performed was not available.

Time from injury to clinic presentation was stratified into three intervals: same-day (0 days), 1–3 days, and >3 days. Delayed presentation was defined as seeking medical care more than 3 days after injury. This threshold was chosen to identify delayed healthcare-seeking behavior.

According to wound severity, the WHO categorizes animal contact into three exposure categories: (i) category I: touching or feeding animals, animal licks on intact skin; (ii) category II: nibbling of uncovered skin, minor scratches or abrasions without bleeding; (iii) category III: single or multiple transdermal bites or scratches, contamination of mucous membrane or broken skin with saliva from animal licks, exposures due to direct contact with bats (1).

Animal ownership status was classified as domestic, wild/stray, rabid or suspected rabid, and unknown. Rabid or suspected rabid animals were defined based on clinical records, including patient report and clinician assessment at the time of presentation, without laboratory confirmation. “Unknown” refers to cases where animal ownership could not be determined.

The species of injurious animal was categorized—based on frequency of involvement in bite incidents—as dog, cat, rodent, rabbit, and other (e.g., ferret, livestock, or unidentified species). Comparisons between groups were performed descriptively, with chi-square tests applied where appropriate, with a significance threshold of *P* < 0.05.

Cases were stratified by month of clinic presentation. Consistent with the climatological seasons in Hangzhou, calendar months were assigned to seasons as follows: spring (March–May), summer (June–August), autumn (September–November), and winter (December–February). Seasonal patterns were described based on monthly case distribution.

### 2.4 Statistical analysis

All data were meticulously entered and structured in Microsoft Excel (Microsoft Office 365), and statistical analyses—including chi-square tests for categorical variables—were conducted using R (version 4.5.2).

Descriptive statistics were used to summarize patient and injury characteristics. Given the large sample size, statistical significance testing was not performed for simple frequency distributions.

Multivariable logistic regression analyses were conducted to:

(1) Identify factors associated with delayed presentation (>3 days), and.
(2) Examine differences between dog- and cat-related injuries.

Covariates included in the multivariable models were selected *a priori* based on clinical relevance and prior literature, rather than statistical screening procedures. Reference categories were selected based on clinical interpretability for each model. Cases with unspecified or heterogeneous injury sites (“other”) were excluded from multivariable analyses to ensure interpretability.

### 2.5 Ethics statement

This study adhered to the ethical principles outlined in the Declaration of Helsinki. Ethical approval was obtained from the Ethics Committee of the Hangzhou Center for Disease Control and Prevention (Hangzhou Health Supervision Institution) under protocol number 2026–46. All personal identifiers were removed prior to analysis, and only anonymized, aggregated data were used.

## 3 Results

### 3.1 Time distribution

**Figure 1:**
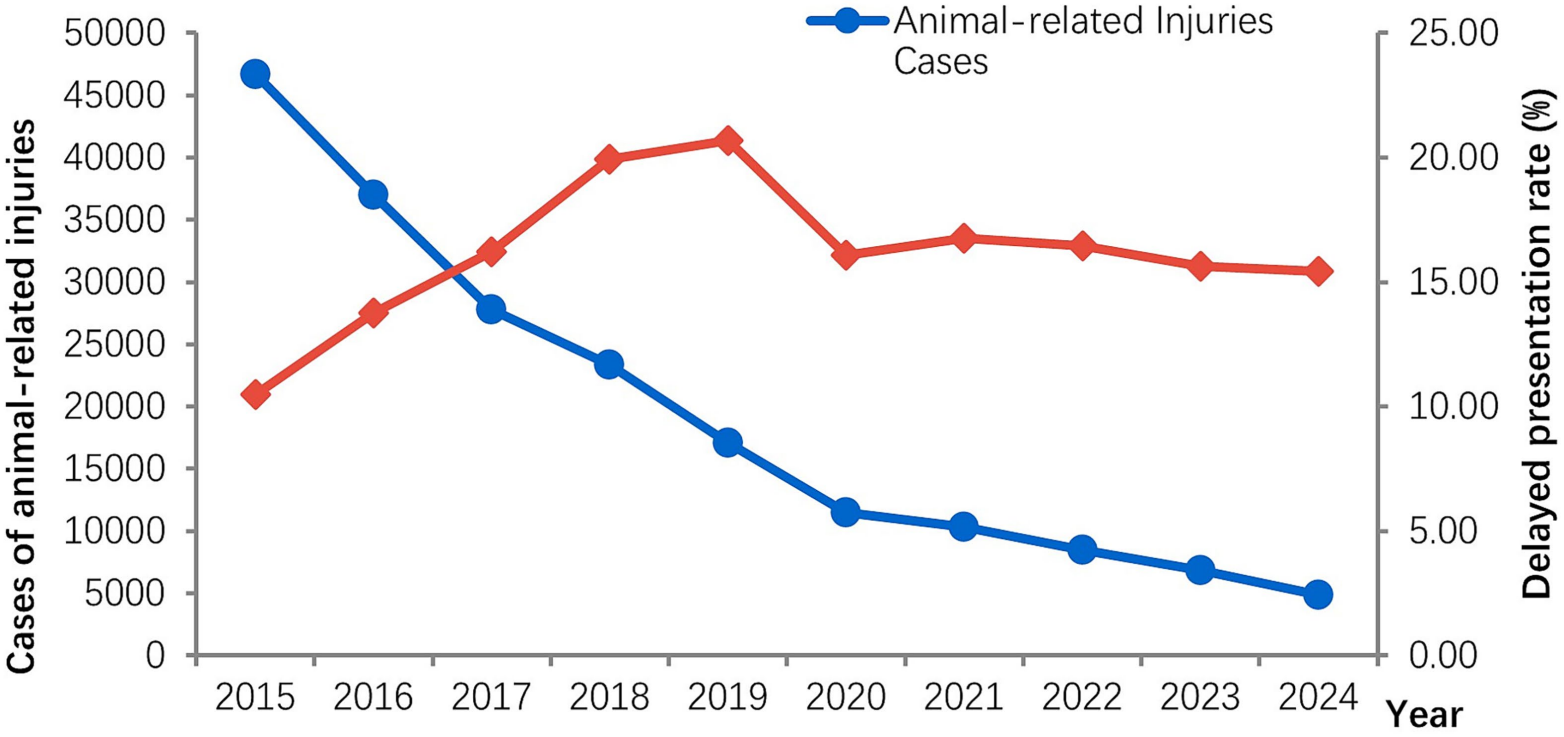
Annual number of clinic presentations and proportion of delayed presentation (>3 days), 2015–2024.

Between January 2015 and December 2024, a total of 193,849 animal-related injury cases in Hangzhou were enrolled in this investigation. A declining trend in the annual number of cases was observed over the study period. However, the delayed presentation rate for over 3 days increased steadily from 10.48% in 2015 to a peak of 20.67% in 2019, subsequently declining and stabilizing at approximately 16% through subsequent years (see Figure 1).

**Figure 2:**
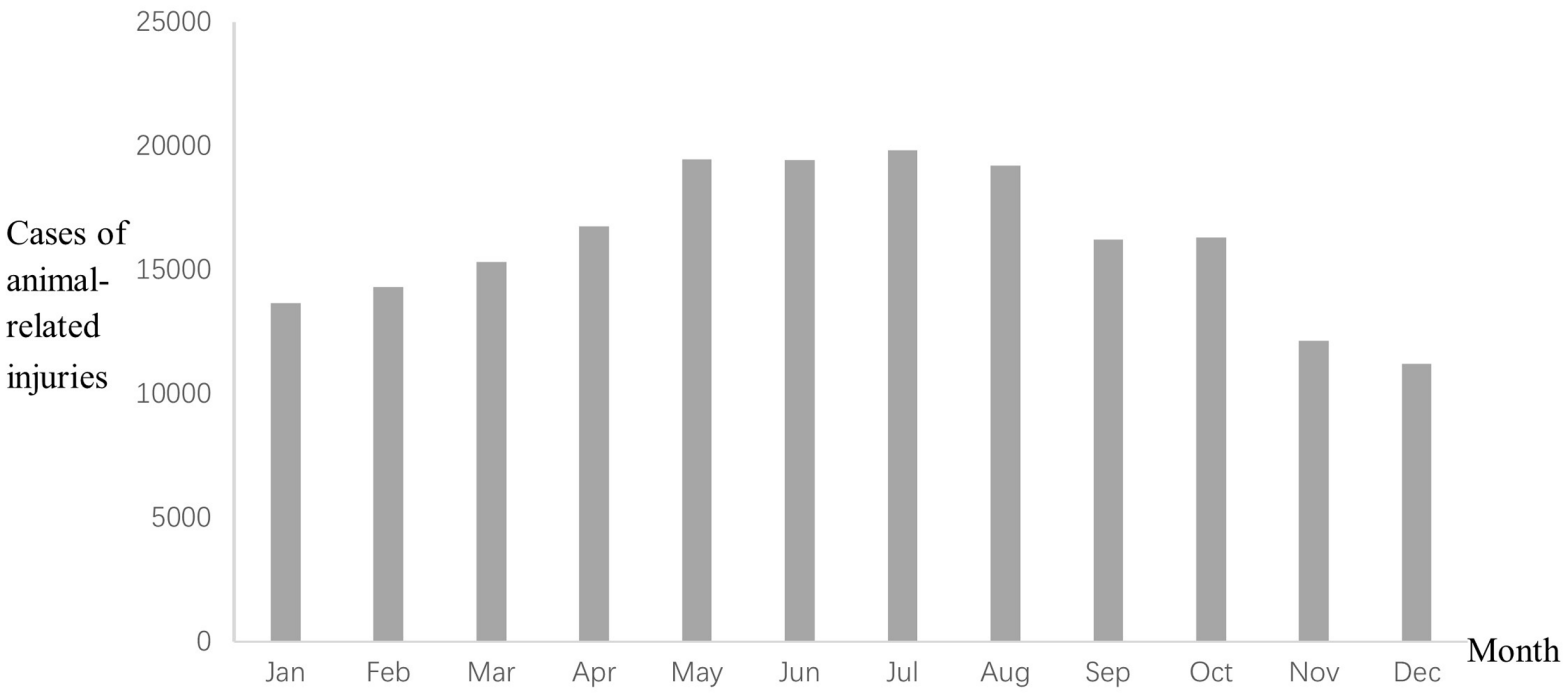
Monthly distribution of animal-related injury presentations at the study clinic, 2015–2024.

The temporal analysis revealed distinct seasonal patterns in animal-related injuries. The summer season (June to August) recorded the highest frequency of incidents, accounting for 58,471 cases (30.16%), with an average of 19,490 cases per month. This was followed by spring (March to May) with 51,513 cases (26.57%), autumn (September to November) with 44,665 cases (23.04%), and winter (December to February) recording the lowest number of cases at 39,200 (20.22%). On a monthly basis, July exhibited the peak incidence with 19,830 cases, while December showed the minimum at 11,222 cases. Monthly case counts ranged from 11,222 to 19,830, with a mean of 16,154 cases per month. The differences in case numbers across seasons demonstrated statistical significance (*χ*^2^ = 8,527.34; *p* < 0.001) (see Figure 2).

### 3.2 Descriptive results

Among 193,849 clinic presentations, females accounted for 55.9% of cases. The largest proportion was observed in the 16–30 age group (36.2%), followed by 31–45 (20.2%) and 46–60 years (19.2%). Upper extremities were the most frequently involved injury site (61.1%), followed by lower extremities (27.5%). Most patients reported both self-treatment and subsequent clinic treatment (61.9%). More than half of patients presented on the same day (53.3%), while 11.4% presented more than 3 days after injury. Category II exposures accounted for the majority of cases (83.8%). Most injuries were caused by domestic animals (82.5%), and dogs and cats together accounted for over 90% of cases (see Table 1).

**Table 1:** Characteristics of animal-related injury presentations at a rabies vaccination clinic in Hangzhou (*N* = 193,849).

Characterisic	Variable	Level	No. of cases	Percentage (%)
Demographic characteristics	Gender	Female	108,324	55.88
		Male	85,525	44.12
	Age (years)	≤15	30,351	15.66
		16–30	70,089	36.16
		31–45	39,145	20.19
		46–60	37,143	19.16
		>60	17,121	8.83
Injury characteristics	Injury Site	Head/neck/face	5,229	2.70
		Upper limb	118,473	61.12
		Trunk	3,994	2.06
		Lower limb	53,388	27.54
		Others	12,765	6.59
	Wound Management	Self-treatment and Hospital treatment	120,069	61.94
		Hospital treatment only	32,881	16.96
		Self-treatment only	40,862	21.08
		No Treatment	37	0.02
	Time from Injury to Clinic Treatment (days)	0	103,244	53.26
		1–3	68,480	35.33
		>3	22,125	11.41
	Animal Contact Category	Grade I	1,623	0.84
		Grade II	162,389	83.77
		Grade III	29,837	15.39
Animal characteristics	Animal Ownership Status	Domestic	159,959	82.52
		Rabid/Suspicious Rabid	437	0.23
		Wild/Stray	12,522	6.46
		Unknown	20,931	10.80
	Injurious Animal	Dog	127,659	65.85
		Cat	53,574	27.64
		Rat	7,957	4.10
		Rabbit	2,140	1.10
		Others	2,519	1.30

### 3.3 Delayed presentation multivariable analysis

To identify the key factors influencing delayed medical treatment (>3 days) in patients injured by animals, we conducted a multivariate logistic regression analysis in Table 2, taking those who sought medical treatment within 3 days as the reference group.

**Table 2:** Multivariable logistic regression analysis of factors associated with delayed presentation (>3 days).

Factors	*B*	S. E.	Wald	*p*-value	Odds ratio (95% CI)
Gender					
Male (Ref.)					
Female	0.032	0.015	4.595	0.032**	1.03 (1.00, 1.06)
Age (y)					
>60 (Ref.)					
≤15	−0.342	0.032	115.945	<0.001**	0.71 (0.67, 0.76)
16–30	−0.247	0.029	70.424	<0.001**	0.78 (0.74–0.83)
31–45	−0.153	0.032	23.466	<0.001**	0.86 (0.81–0.91)
46–60	0.006	0.031	0.036	0.849	1.01 (0.95–1.07)
Injury site					
Lower Limb (Ref.)					
Head, neck or Face	−0.695	0.05	192.73	<0.001**	0.50 (0.45–0.55)
Upper Limb	0.017	0.016	1.141	0.285	1.02 (0.99–1.05)
Trunk	0.028	0.051	0.302	0.583	1.03 (0.93–1.14)
Animal contact category					
Grade II (Ref.)					
Grade I	0.481	0.181	7.011	0.008**	1.62 (1.15, 2.35)
Grade III	1.66	0.035	2,239	<0.001**	5.29 (4.94, 5.67)
Animal category					
Domestic (Ref.)					
Rabid/Suspicious Rabid	0.829	0.022	1461.322	<0.001**	2.29 (2.20–2.39)
Wild/Stray	1.276	0.023	2969.918	<0.001**	3.58 (3.42–3.75)
Unknown	−2.484	1.29	3.707	0.054	0.08 (0.00–0.97)
Injurious animal					
Dog (Ref.)					
Cat	0.471	0.166	8.026	0.005**	1.60 (1.17–2.25)
Rat	1.15	0.046	636.033	<0.001**	3.16 (2.89–3.46)
Rabbit	1.42	0.034	1761.186	<0.001**	4.14 (3.87–4.42)
Other					

CI, confidence interval; **p < 0.05.

Female sex was associated with a slightly increased likelihood of delayed presentation (OR = 1.03, 95% CI: 1.00–1.06), although the magnitude of this effect was small. Compared with individuals aged >60 years, all younger age groups were significantly less likely to experience delayed presentation, including those aged <15 years (OR = 0.71, 95% CI: 0.67–0.76), 16–30 years (OR = 0.78, 95% CI: 0.74–0.83), and 31–45 years (OR = 0.86, 95% CI: 0.81–0.91), while no significant difference was observed for those aged 46–60 years.

Injuries involving the head and face were associated with a lower likelihood of delayed presentation (OR = 0.50, 95% CI: 0.45–0.55), whereas injuries to the upper limbs and trunk did not differ significantly from lower limb injuries. Using WHO Category II exposure as the reference group, both Category I (OR = 1.62, 95% CI: 1.15–2.35) and Category III exposures (OR = 5.29, 95% CI: 4.94–5.67) were associated with higher odds of delayed presentation, with a substantially stronger association observed for Category III exposures.

Compared with dog-related injuries, injuries caused by cats (OR = 1.60, 95% CI: 1.17–2.25), rodents (OR = 3.16, 95% CI: 2.89–3.46), and rabbits (OR = 4.14, 95% CI: 3.87–4.42) were associated with higher odds of delayed presentation. Additionally, injuries caused by rabid or suspected rabid animals (OR = 2.29, 95% CI: 2.20–2.39) and wild animals (OR = 3.58, 95% CI: 3.42–3.75) were more likely to be associated with delayed presentation compared with those caused by domestic animals.

### 3.4 Comparison of dog-related and cat-related injuries

Dogs and cats accounted for the majority of animal-related injury presentations and were therefore compared. Among 181,233 cases, dog-related injuries comprised 70.4%, while cat-related injuries accounted for 29.6%. Differences in demographic distribution were observed between the two groups. Cat-related injuries were more frequently reported among females (31,773 cases, 59.31%), whereas dog-related injuries accounted for a larger share of cases across most age groups. The distribution of injured areas also varies. Injuries related to cats occur more frequently in the upper limbs (39,394 cases, accounting for 73.53%), while for injuries related to dogs, the upper limbs account for 55.23% and the lower limbs 32.69%. Most injuries in both groups involved domestic animals, although this proportion was higher among dog-related injuries (89.74% vs. 71.40%) (see Table 3).

**Table 3:** Variables of dog- and cat-related injuries presentations (*N* = 181,233).

Variable	Level	Dog injury		Cat injury	
		No. of cases	Percentage (%)	No. of cases	Percentage (%)
Gender	Female	69,743	54.63	31,773	59.31
	Male	57,916	45.37	21,801	40.69
Age (y)	≤15	20,468	16.03	6,810	12.71
	16–30	40,154	31.45	25,878	48.30
	31–45	26,123	20.46	10,328	19.28
	46–60	27,955	21.90	7,424	13.86
	>60	12,959	10.15	3,134	5.85
Injury site	Head/neck/ face	3,714	2.91	2028	3.79
	Upper limb	70,506	55.23	39,394	73.53
	Trunk	2,230	1.75	1,525	2.85
	Lower limb	41,738	32.69	10,426	19.46
	Others	9,471	7.42	201	0.38
Animal contact category	Grade I	378	0.30	70	0.13
	Grade II	108,994	85.38	44,857	83.73
	Grade III	18,287	14.32	8,647	16.14
Animal ownership status	Domestic	114,565	89.74	38,250	71.40
	Rabid/suspicious rabid	251	0.20	129	0.24
	Wild/stray	1,126	0.88	8,247	15.39
	Unknown	11,717	9.18	6,948	12.97

To comparatively characterize injury patterns associated with cat and dog exposures, we performed a multivariable logistic regression analysis using dog-related injuries as the reference category. In multivariable analysis, cat-related injuries were more likely to occur among females (OR 1.227, 95% CI 1.200–1.255) and younger individuals, particularly those aged 16–30 years (OR 2.752, 95% CI 2.630–2.881). Cat-related injuries were also more likely to involve upper body sites, including the upper extremities (OR 2.132, 95% CI 2.076–2.189) and head/face (OR 2.285, 95% CI 2.140–2.440). No significant difference was observed for Category II exposures. Compared with domestic animals, wild cats cause significantly more injuries than wild dogs among wild animals (OR 19.863, 95% CI 18.648–21.176) (see Table 4).

**Table 4:** Multivariable logistic regression analysis comparing cat-related vs. dog-related injuries.

Factors	*B*	S. E.	Wald	*p*-value	Odds ratio (95% CI)
Gender					
Male (Ref.)					
Female	0.205	0.011	320.037	<0.001**	1.227 (1.200, 1.255)
Age (y)					
>60 (Ref.)					
≤15	0.353	0.026	179.870	<0.001**	1.424 (1.352, 1.499)
16–30	1.012	0.023	1907.651	<0.001**	2.752 (2.630, 2.881)
31–45	0.544	0.025	473.056	<0.001**	1.723 (1.641, 1.810)
46–60	0.178	0.025	51.481	<0.001**	1.195 (1.138, 1.255)
Injury site					
Lower Limb (Ref.)					
Head, neck or Face	0.826	0.033	612.456	<0.001**	2.285 (2.140, 2.440)
Upper Limb	0.757	0.013	3151.909	<0.001**	2.132 (2.076, 2.189)
Trunk	1.097	0.037	895.839	<0.001**	2.996 (2.788, 3.219)
Animal contact category					
Grade III (Ref.)					
Grade I	−2.416	0.158	233.117	<0.001**	0.089 (0.065, 0.121)
Grade II	0.009	0.017	0.324	0.569	1.010 (0.977, 1.043)
Animal category					
Domestic (Ref.)					
Rabid/Suspicious Rabid	0.265	0.112	5.634	0.018 **	1.303 (1.045, 1.619)
Wild/Stray	2.989	0.032	8493.541	<0.001**	19.863 (18.648, 21.176)
Unknown	0.521	0.017	949.431	<0.001**	1.684 (1.629, 1.741)

CI, confidence interval; **p < 0.05.

### 3.5 Injuries by rabid or suspected rabid animals

A total of 437 cases involved rabid or suspected rabid animals. Most cases were classified as Category II exposures, and the majority of injuries involved the upper extremities. Domestic animals accounted for a substantial proportion of cases, although cases involving stray or wild animals were also observed. These findings should be interpreted cautiously given the classification of rabid or suspected rabid animals was based on clinical records without laboratory confirmation (see Table 5).

**Table 5:** Descriptive characteristics of cases involving rabid or suspected rabid animals (*N* = 437).

Variable	Level	No. of cases	Percentage (%)
Gender	Female	257	58.81
	Male	180	41.19
Age (y)	≤15	56	12.81
	16–30	179	40.96
	31–45	92	21.05
	46–60	79	18.08
	>60	31	7.09
Injury site	Head/neck/face	16	3.66
	Upper limb	307	70.25
	Trunk	5	1.14
	Lower limb	105	24.03
	Others	4	0.92
Injurious animal	Dog	251	57.44
	Cat	129	29.52
	Rat	32	7.32
	Rabbit	2	0.46
	Others	23	5.26
Animal contact category	Grade I	0	0.00
	Grade II	268	61.33
	Grade III	169	38.67

## 4 Discussion

This study provides a large-scale descriptive overview of animal-related injury presentations at a single high-volume clinic. Distinct from population-level epidemiological studies, our study specifically examines patterns of medical resource utilization and case mix composition. A total of 193,849 clinical cases were included in this study from 2015 to 2024, revealing distinct time trends, demographic attributes, clinical characteristics, and animal sources. This study further examined determinants of delayed presentation for medical care and compared demographic and clinical profiles between patients with cat-associated versus dog-associated injuries. These findings may support clinicians in optimizing case identification and risk stratification, and provide evidence-based support for epidemiologists in formulating precise health education strategies.

With respect to temporal patterns, summer months (June through August) exhibited the highest frequency of animal-related injuries, comprising 30.16% of annual cases. This observation aligns with numerous domestic and international investigations. Research from Beijing’s Haidian District spanning 2011–2020 (16) likewise revealed comparable temporal distribution patterns, with June through August representing the peak season. Yılmaz et al. (10) similarly documented in their Turkish single-clinic investigation that dog-related injuries reached their zenith in summer. The elevated occurrence of animal-related injuries during summer months may be associated with the following factors: firstly, increased summer temperatures can trigger behavioral modifications in animals (particularly canines), including heightened irritability and aggression (11); secondly, individuals wear more minimal clothing during summer and participate more frequently in outdoor pursuits, amplifying opportunities for animal encounters (26); thirdly, extended daylight hours in summer entail greater outdoor exposure time.

The declining trend in annual case numbers should be interpreted with caution. As this was a single-center study conducted at a major rabies vaccination clinic, temporal changes may reflect shifts in healthcare utilization, service availability, or redistribution of patients across institutions, rather than a true change in incidence. In fact, the number of rabies clinic increased from 4 in 2015 to over 70 in 2024 in Hangzhou, which is 18-fold expansion over the decade.

In terms of age distribution, the 16–30 age cohort exhibited the greatest number of animal-related injury cases (70,089 cases, 36.2%), succeeded by the 31–45 age group (20.2%) and the 46–60 age group (19.2%). This finding corresponds with multiple investigations. The Beijing Haidian District study (16) similarly ascertained that young and middle-aged adults represented the high-risk demographic for animal-related injuries. Wang et al. (15), in their descriptive epidemiological examination of animal-related injuries in a single clinic also in Hangzhou, likewise documented comparable age distribution patterns. The more observed cases among young and middle-aged adults may be attributed to their frequent social engagements, greater outdoor pursuits, and expanded opportunities for animal contact. Of note, the ≤15 age cohort represented 15.7% (30,351 cases), and although this proportion falls below that of the young adult cohort, the absolute figure remains considerable. Children and adolescents constitute an important part presenting to our clinic for animal-related injuries, which may be associated with children’s smaller physical stature, diminished self-protection capabilities, and insufficient judgment of animal behavior. Patterson and associates (27), in their systematic review of pediatric dog-related injuries in the United States, determined that children represent a particularly vulnerable population for dog-related injuries. Essig et al. (28), in their systematic review and meta-analysis, additionally substantiated the association between canine breed and risk of facial dog-related injuries among children. Although animal-related injury exposure among children may be lower than among adults, the fatality risk is greater due to factors such as delayed wound management and immunization, meriting particular attention.

With regard to injury characteristics, upper extremities emerged as the most frequently injured anatomical site, representing 61.1% (118,473 cases), followed by lower extremities (27.5%) and head/face/neck regions (2.7%). This finding demonstrates substantial consistency with domestic and international research. Li et al. (19), in their investigation in Central China, ascertained that upper extremities constituted the most prevalent site of animal-related injuries. Ehrhard et al. (29), in their study at a Swiss tertiary emergency department, likewise documented that upper extremities represented the most frequent site of animal-related injuries. The elevated proportion of upper extremity injuries may reflect two interrelated behavioral mechanisms: first, the innate protective reflex of using the arms and hands to shield the head, face, and torso during sudden animal encounters; second, recurrent exposure patterns inherent in routine human–pet interactions, such as lifting, restraining, or comforting animals, which place the upper extremities at heightened mechanical risk. It is worth noting that although head and facial injuries constitute merely 2.7%, owing to the abundant vasculature of the head and face and proximity to the central nervous system, once injured, serious consequences may ensue (27). The present investigation revealed that WHO Category II exposure accounted for 83.77% and WHO Category III exposure represented 15.39%, a distribution analogous to other studies. Wang et al.(15), in a study of animal bite patients in China, reported that Category II exposures accounted for the largest proportion of cases. In contrast, Kumar et al. (30) from India found that 83.7% of bite incidents were classified as Category III—highlighting marked geographical divergence in exposure severity distribution. However, the comparatively diminished proportion of Category III exposure in this investigation may reflect a higher percentage of minor cases, or possibly that some severe cases were directly referred to tertiary hospitals and not incorporated in our clinic surveillance.

Although post-exposure prophylaxis (PEP) is central to rabies prevention, detailed data on PEP initiation, completion, and appropriateness were not available in this dataset. Therefore, this study does not directly evaluate PEP practices. Instead, time to presentation was used as a proxy indicator of healthcare-seeking behavior, which may partially reflect the timeliness of post-exposure care. Delayed presentation (>3 days) observed in a subset of patients highlights potential gaps in timely healthcare access or awareness.

The majority of patients successfully accessed medical care promptly, reflecting these patients’ satisfactory awareness of animal-related injury hazards and excellent healthcare accessibility. Nevertheless, in our study, more than 10% of patients deferred medical care for more than 3 days, which may amplify the risk of rabies and other infections. Liu et al. (21) ascertained that postponement of post-exposure prophylaxis (PEP) among animal bite victims in China persist as a substantial public health concern, potentially influenced by sociodemographic determinants including educational attainment and sex. de Feij et al. (31) documented postponements in post-exposure prophylaxis (PEP) during international travel, indicating that even when medical resources are adequate, delays in accessing care nevertheless transpire.

Multivariate analysis revealed that younger individuals were less likely to experience delayed presentation compared with older adults, which may reflect differences in health awareness, caregiver involvement, or healthcare access patterns. Three interrelated factors are likely to contribute to this pattern. First, inadequate risk perception, particularly about rabies transmission risk, may cause older individuals to underestimate the seriousness of animal bites and exhibit a more relaxed attitude toward medical care. Secondly, the physical condition of older adults may prevent them from obtaining medical care services in a timely manner. Thirdly, although most people over 60 have pensions, they still consider the cost issue, which makes out-of-pocket medical expenses a serious obstacle to seeking medical care in a timely manner—a finding consistent with prior Chinese studies identifying financial constraints as a key determinant of treatment delay (21).

Additionally, injury location independently predicted delay: Injuries involving the head and face were associated with more prompt care-seeking, likely due to higher perceived severity and potential cosmetic concerns. When using Category II exposure as the reference group, both Category I and Category III exposures were associated with increased odds of delayed presentation, with a particularly strong association observed for Category III exposures. Although severe exposures (Category III) are typically associated with increased perceived risk and urgency, our findings suggest that, within a clinic-based dataset, the observed time to presentation may also be influenced by referral patterns and prior healthcare utilization. Patients with severe (Category III) injuries may initially seek care at emergency departments or surgical facilities for wound management and subsequently present to rabies vaccination clinics, resulting in a longer recorded time to presentation in this setting. In contrast, Category II exposures, which represent the most common and clinically typical injury pattern, are more likely to be managed directly at rabies clinics without prior referral, leading to shorter observed delays. Therefore, the observed association should be interpreted in the context of healthcare system pathways rather than differences in patient awareness alone. The relatively large effect size observed for Category III exposure (OR >5) further underscores the substantial differences in care pathways and clinical management across exposure categories, rather than indicating implausible behavioral patterns.

In addition, Injuries caused by non-dog species, including cats, rodents, and rabbits, were associated with increased odds of delayed presentation. This may reflect lower perceived rabies risk or differences in exposure context, leading to delayed recognition of the need for post-exposure prophylaxis. Similarly, injuries caused by rabid or suspected rabid animals and wild animals were associated with increased delay, which may reflect more complex exposure circumstances or delayed recognition of risk.

Regarding specific animal species, dogs accounted for 65.8% (127,659 cases), cats for 27.6% (53,574 cases). These findings are consistent with previous studies conducted in China and internationally. The China animal rabies surveillance report 2004–2024 (8, 12) demonstrates that dogs represent the primary source of animal-related injuries and rabies transmission in China. Miao et al. (14) emphasized that the challenges in regulating animal rabies in China predominantly derive from inadequate canine vaccination coverage and complexities in managing stray animals. Ren et al. (18), in their early investigation of human rabies in Zhejiang Province, established that dogs function as the principal host for rabies virus transmission. On a global scale, Hampson et al. (2) projected that approximately 59,000 individuals succumb to rabies annually worldwide, with the preponderance transmitted by dogs. Significantly, cat-related injuries comprised 27.6% in this investigation, a proportion that warrants attention.

We performed a comparative analysis of cat- and dog-associated injuries. Multivariable analysis showed that cat-related injuries were more likely to occur among females and younger individuals after adjustment for other variables. Furthermore, cat-related injuries were more frequently associated with upper-body injury sites. These findings suggest that the observed differences between cat- and dog-related injuries are not fully explained by demographic composition. For the female patients presenting for animal-related injury care, cat-inflicted injuries is significantly higher compared with those injured by dogs, which is a pattern consistently reported across multiple population-based studies (32). This sex disparity may be plausibly attributed to behavioral and sociocultural factors that could not be directly measured in the present study. For instance, women may be more likely to have closer and more frequent physical contact with cats (e.g., holding, hand-feeding), which could increase the risk of bites and scratches (33). In addition, women may be more inclined to seek medical attention and report animal-related injuries, whereas men may underreport such events (34).

Cat-related injuries were observed across all age groups, suggesting a relatively broad distribution compared with dog-related injuries. In contrast, reports from the United States show that dog bites are more common in children and young individuals, especially children under 9 years old who are particularly vulnerable to dog bites (35). This difference may reflect differences in pet ownership culture and contact patterns across different regions.

Anatomical injury location also differed markedly: cat injuries were more common than dog injuries for upper body injuries. This may be related to differences in interaction patterns between humans and animals. Research reports that cat bites mainly occur in the upper limbs, followed by the face; while dog bite site distribution is more diverse (32). Previous studies have reported that cat-related injuries more commonly involve upper limbs and face, whereas dog-related injuries tend to have a more diverse distribution of injury sites (32, 36). These findings may help contextualize the patterns observed in this study.

Injuries from stray cats vastly outnumbered those from stray dogs, may reflect differences in urban animal management practices. Previous studies have suggested that differences in stray animal populations may be influenced by variations in urban animal management practices (37). This may provide context for the higher proportion of stray cat-related injuries observed in this study.

The present investigation specifically examined 437 cases of injuries by rabid or suspected rabid animals, the highest-risk exposure category. Among these cases, females represented 58.8%, the 16–30 age cohort constituted 41.0%, and upper extremity injuries comprised 70.3%. Dogs served as the primary injuring animals (57.4%), succeeded by cats (29.5%). In WHO classification, Category II exposure accounted for 61.3% and Category III exposure for 38.7%. These findings imply that even for injuries by rabid or suspected rabid animals, Category II exposure nevertheless constitutes the majority. The interpretation of this finding is limited, as the classification of rabid or suspected rabid animals was based on clinical records without laboratory confirmation. Nonetheless, the proportion of category III exposure was as high as 38.7%, significantly higher than the 15.39% in the general population. This suggests that when clinical doctors assess suspected rabies animal bite cases, they tend to adopt a more conservative exposure classification strategy, that is, they prefer to categorize them as high-risk exposure. Chen et al. (38), in their examination of epidemiological characteristics of human rabies in Chongqing from 2016 to 2024, determined that rabies cases were predominantly male, differing from this study’s finding of elevated female proportions. This may indicate that there are differences in the distribution of the population between animal bites and laboratory-confirmed rabies cases.

The present investigation possesses certain limitations. Firstly, this investigation relied upon passive surveillance system data, which may entail underreporting and reporting bias; secondly, this investigation incorporated cases exclusively from one clinic in Hangzhou, and extrapolation of results to other regions should be executed judiciously; thirdly, incomplete information on certain variables may affect the comprehensiveness of analysis; finally, this investigation constituted a descriptive epidemiological study and could not thoroughly elucidate the specific mechanisms and risk factors of animal-related injuries. Despite these limitations, this investigation encompassed a substantial sample size, extended study duration, and relatively elevated data quality, establishing results of considerable reference value.

## 5 Conclusion

This large retrospective analysis of animal-related injury presentations over a 10-year period provides a detailed description of patterns observed in a high-volume rabies vaccination clinic in Hangzhou. The findings indicate that demographic characteristics, exposure severity, and animal species are associated with variations in healthcare-seeking behavior and injury patterns. Delayed presentation was more common among younger individuals and those with less severe exposures, suggesting gaps in risk perception and health awareness. Differences between dog- and cat-related injuries persisted after adjustment for potential confounders, highlighting distinct exposure contexts and interaction patterns. Given the clinic-based nature of the data, these findings reflect healthcare utilization patterns rather than population-level incidence. Nevertheless, they provide useful insights for public health practice, particularly in guiding health education efforts aimed at promoting timely medical evaluation following animal exposure. Future studies incorporating population-based data and detailed post-exposure prophylaxis information are warranted.

## Data Availability

The original contributions presented in the study are included in the article/supplementary material, further inquiries can be directed to the corresponding author.
